# Editorial: Physical activity, sports and health: reflections and challenges based on sustainability

**DOI:** 10.3389/fspor.2025.1547197

**Published:** 2025-01-20

**Authors:** Francisco José Gondim Pitanga, Victor Keihan Rodrigues Matsudo, Dartagnan Pinto Guedes

**Affiliations:** ^1^ Multidisciplinary Institute of Rehabilitation and Health, Federal University of Bahia, Salvador, Brazil; ^2^ Studies Center of the Physical Fitness Research Laboratory from São Caetano do Sul, São Caetano do Sul, São Paulo, Brazil; ^3^ Health Sciences Center, State University of Northern Paraná, Jacarezinho, Brazil

**Keywords:** physical activity, sustainability, health, SDGs (sustainable development goals), physical exercise

**Editorial on the Research Topic**
Physical activity, sports and health: reflections and challenges based on sustainability

The benefits of physical activity for the health of individuals and populations have been considered for a long time. Initially, and until very recently, the emphasis was on the main positive results from the perspective of cardiovascular, metabolic and mental health (1, 2), as well as in the prevention and treatment of cancer (3). Regarding the immune system, although since the 1980s, many researchers have been examining its relationship with physical activity, with the arrival of the Covid-19 pandemic research on this topic has increased significantly (4). In this sense, it becomes very important to understand how physical activity can impact the prevention and treatment of both communicable and non-communicable diseases, and how the sustainable development goals (SDGs) can impact this context.

In this perspective, in 2015, all member countries of the United Nations (UN) (5) adopted and began to share the 17 SDGs: (1) No poverty; (2) Zero hunger; (3) Good health and well-being; (4) Quality education; (5) Gender equality; (6) Clean water and sanitation; (7) Affordable and clean energy; (8) Decent work and economic growth; (9) Industry, innovation and infrastructure; (10) Reduction inequalities; (11) Sustainable cities and communities; (12) Responsible consumption and production; (13) Climate action; (14) Life below water; (15) Life on land; (16) Peace, justice and strong institutions; (17) Partnerships for the goals.

Although different experts are trying to identify SDGs that may be linked to physical activity (6, 7), we have difficulty understanding the relevance of this unbridled search, especially from the perspective of effective contributions to increasing the practice of physical activity by the population and its respective health benefits. Thus, in the editorial, we intend to focus on commenting on the SDGs that are directly linked to the manuscripts that were published in this special issue.

Thus, we will initially look at the third objective of the SDGs, “Good health and well-being”, which points to two indicators that seem to be directly linked to physical activity, namely: by 2030, end the epidemics of AIDS, tuberculosis, malaria, and neglected tropical diseases, and combat hepatitis, waterborne diseases, and other communicable diseases; and reduce premature mortality from noncommunicable diseases by one third through prevention and treatment; and promote mental health and well-being. In both indicators, we can see that physical activity can play an important role in the prevention and treatment of both communicable and noncommunicable diseases.

In this sense, the study by Xu et al. analyzed the complex relationship of factors that affect the level of participation in physical fitness activities in China, finding as main results that public financial support for mass sports and the provision of sports venues are the main conditions for improving the level of participation in physical fitness activities. The authors also highlight the importance of this participation in the prevention of both infectious and chronic-degenerative diseases.

From another perspective, the study by Gomes et al. offers an overview of relevant research on the relationship between physical activity or exercise and oral diseases. In this work, the authors draw attention to the need for greater attention to dysfunctional habits that can contribute to premature tooth wear, as well as to oral inflammatory diseases that can have systemic implications and influence the practice of physical activity.

Still regarding the third objective of SDGs, specifically the promotion of mental health, the manuscript by Jiménez-Maldonado et al. points out that physical activity performed through combined exercise (i.e., aerobic plus strength training) has a significant positive effect on general cognition and executive function, although the authors report that there are few studies on this topic in Latin American and Caribbean countries. The authors suggest the urgent need for research to identify the feasibility of physical exercise interventions to enhance cognitive abilities in older adults in these regions.

Regarding the eighth objective of SDGs “Decent work and economic growth”, although there is no direct association with physical activity, the manuscript by Abe et al. shows us that exercise-based physical activity is broadly and consistently associated with the lowest level of stress responses triggered by moderate/vigorous work-related physical activity.

Finally, regarding the eleventh objective of SDGs “Sustainable cities and communities”, two aspects draw attention to the possibility of links with physical activity, including active commuting and the practice of physical activity in the local community itself. This would be possible with the construction of adequate spaces for physical activity in sustainable cities and communities.

In this sense, the study by Barbosa et al. demonstrated that performing physical exercises in community facilities improved the quality of life and physical activity levels of older adults. The authors also observed that supervised training was particularly advantageous for improving functional capacity.

On the other hand, studies indicate that we are experiencing a true pandemic of physical inactivity. Recent data from a global survey indicate that the prevalence of insufficient inactivity increased between 2000 and 2022 (8), a fact that generates inestimable losses for public health with costs that could be minimized if people and populations became more physically active. Current figures represent a great challenge for global societies in terms of proposing public policies that can increase the practice of physical activity at a global level. Specifically in Brazil, an interesting proposal was the use of Mobile Management of the Ecological Model (9) by the Agita São Paulo program, which led to a significant reduction of insufficiently active from 39% to 14% and increased levels of physical activity (regularly active from 44% to 62%) in the state of São Paulo (10).

However, at the present time, it is clear that physical activity, as a human behavior, is going through difficult times and urgently needs to be consolidated as an accessible behavior for a greater number of people around the world. The same global survey data cited in the previous paragraph show us a bleak reality for achieving the goal of reducing global physical inactivity by at least 10%. Only two regions, Oceania and Sub-Saharan Africa, are on track to reach the 2030 target, while other regions and most countries will not reach this goal if current trends continue.

We believe that public policies to promote physical activity should be based on more concrete procedures so that goals can be achieved. Thus, paraphrasing the Canadian thinker “Jordan Peterson” (11), one more reflection is necessary: initially, it would not be advisable to tidy up our house (physical activity) and then try to change the world (sustainability) through physical activity. This is the reflection and the challenge.
